# Resilience and CVD-protective Health Behaviors in Older Women: Examining Racial and Ethnic Differences in a Cross-Sectional Analysis of the Women’s Health Initiative

**DOI:** 10.3390/nu12072107

**Published:** 2020-07-16

**Authors:** Sparkle Springfield, FeiFei Qin, Haley Hedlin, Charles B. Eaton, Milagros C. Rosal, Herman Taylor, Ursula M. Staudinger, Marcia L. Stefanick

**Affiliations:** 1 Parkinson School of Health Sciences and Public Health, Department of Public Health, Loyola University Chicago, 2160 S N 1st Ave, Maywood, IL 60153, USA; 2 Quantitative Sciences Unit, Stanford University, Alto, CA 94304, USA; fqin@stanford.edu (F.Q.); hedlin@stanford.edu (H.H.); 3 Warren Alpert Medical School, Department of Family Medicine School of Public Health Brown, Providence University, Providence, RI 02912, USA; CEaton@CareNE.org; 4 Department of Population and Quantitative Health Sciences, Medical School of Massachusetts University, Massachusetts University, Worcester, MA 01605, USA; Milagros.Rosal@umassmed.edu; 5 Research Wing Room, Morehouse School of Medicine Cardiovascular Research Institute, Atlanta, GA 30310, USA; htaylor@msm.edu; 6 Columbia Aging Center & Department of Socio medical Science, Mailman School of Public Health, Columbia University, New York, NY 10032, USA; ums2103@cumc.columbia.edu; 7 Stanford Prevention Research Center, Stanford University, Alto, CA 94304, USA; mstefanick@stanford.edu

**Keywords:** resilience, health behaviors, prevention, WHI, lifestyle

## Abstract

Little is known about the relationship between self-reported psychological resilience (resilience) and health behaviors shown to reduce the risk of cardiovascular disease (CVD). This study examines the associations between resilience and CVD-related risk factors, such as diet, smoking, physical activity, sleep, and alcohol consumption among older American women from diverse backgrounds. Methods: A cross-sectional secondary analysis was conducted on 77,395 women (mean age 77 years, Black (*N* = 4475, 5.8%), non-Hispanic white (*N* = 69,448, 89.7%), Latina (*N* = 1891, 2.4%), and Asian or Pacific Islander (*N* = 1581, 2.0%)) enrolled in the Women’s Health Initiative Extension Study II. Resilience was measured using an abbreviated version of the brief resilience scale. Multivariable logistic regression models were used to evaluate the association between resilience and health behaviors associated with risk for CVD, while adjusting for stressful life events and sociodemographic information. To test whether these associations varied among racial/ethnic groups, an interaction term was added to the fully adjusted models between resilience and race/ethnicity. Results: High levels of resilience were associated with better diet quality (top 2 quintiles of the Healthy Eating Index 2015) (OR = 1.22 (95% Confidence Interval (1.15–1.30)), adhering to recommended physical activity (≥ 150 min per week) (1.56 (1.47, 1.66)), sleeping the recommended hours per night (7–9) (1.36 (1.28–1.44)), and moderate alcohol intake (consuming alcoholic drink(s) 1–7 days per week) (1.28 (1.20–1.37)). The observed association between resilience and sleep is modified by race/ethnicity (*p* = 0.03). Conclusion: Irrespective of race/ethnicity, high resilience was associated with CVD-protective health behaviors. This warrants further investigation into whether interventions aimed at improving resilience could increase the effectiveness of lifestyle interventions.

## 1. Introduction

Although there have been steady declines in the incidence and prevalence of cardiovascular disease (CVD) over the past five decades, it remains the leading cause of death among American women [1,2]. Apart from metabolic risk factors (such as a diagnosis of diabetes, hypertension, and/or high cholesterol), poor health behaviors, including an unhealthy diet, smoking cigarettes, physical inactivity [2], and sleeping too much or too little [3] significantly increase the risk of dying from CVD. Despite the growing awareness of how a healthy lifestyle can reduce these risk factors, many women do not adhere to preventative recommendations [4,5].

Tied to this, biological and epidemiological evidence suggests that excessive psychological stress—that taxes or exceeds one’s resources and adaptive capacity—has disruptive effects on cardiovascular health including the diet, physical activity, sleep behavior, and the likelihood that an older woman will adhere to behavioral interventions [6,7,8,9]. This evidence extends to racially and ethnically diverse older female populations [10,11,12] and may be particularly relevant to specific racial/ethnic groups. In particular, Black women face structural and personally mediated racism, and subsequently experience disproportionately higher levels of race-related stress at community and individual levels [13,14]. This is the result of both historic segregation [15] and contemporary discrimination [16], and manifests itself in limited access to health resources, such as full-service supermarkets [17] and green spaces [18]. Furthermore, higher perceived discrimination has been associated with lower engagement in protective health behaviors among Black women [19].

Psychological resilience (hereafter referred to as “resilience”) is defined as the self-reported ability to bounce back from stress. Since it is modifiable and assessable, it may foster protective health behaviors [20], irrespective of one’s social and physical environment [21]. Recent epidemiological studies demonstrate that resilience is positively associated with CVD-protective health behaviors, which include better diet quality [22], non-smoking status [23,24], increased physical activity [25], adequate sleep duration [26,27], and moderate alcohol consumption [28]. It is important to note, however, that these studies have not focused exclusively on older women. There is also limited research on the relationship between resilience and CVD-protective health behaviors in racially and ethnically diverse older women from the general population [19,20].

That being said, Kim et al. [29] used census data to discover that there are “resilient neighborhoods” throughout the United States, where Black residents had substantially lower than expected rates of CVD events. Yet, the team did not evaluate the relationship between individual-level psychological resilience and protective behaviors in older women [29]. Similarly, Saban et al. [30] reported that subjective social status and social support may contribute to perceived resilience among Black women. While social support is a protective factor against CVD and is associated with positive health behaviors, their study did not evaluate perceived resilience and associated health behaviors [30].

More closely aligned with our study objective, Felix et al. [10] examined associations of stress with incident CVD among Black women enrolled in the Women’s Health Initiative (WHI) and tested for effect modification by the brief resilience scale (BRS). They discovered that higher stressful life events were significantly and positively associated with incident CVD. Although resilience did not modify this relationship nor was it associated with CVD, women with higher resilience reported healthier characteristics (i.e., more physical activity and less obesity) compared to women with lower resilience. Considering that the BRS was only conducted once—in 2011—and most of the relevant health behaviors and stressful life events were measured that same year [10], it is necessary to build upon this foundation to evaluate the main effect of resilience health behaviors.

Addressing this gap, our study examines the associations between resilience and the CVD-protective behaviors listed above among racially and ethnically diverse older women in the Women’s Health Initiative, Extension Study II. It is important to frame our contribution in the context of policy and public health interventions, namely, we are evaluating the relationship between resilience and health behaviors as a foundational step to understanding asset-based and strength-based lifestyle intervention approaches, which may be particularly helpful to and relevant for Black women. Because BRS takes an outcome-oriented approach that defines resilience in terms of how well someone bounces back from stress, it may be ideal for intervention purposes [31,32]. Our team hypothesized that high levels of resilience would be significantly associated with more CVD-protective behaviors, after adjusting for stressful life events, sociodemographic characteristics, and additional CVD risk factors. Additionally, we hypothesized that these associations would differ by race/ethnicity.

Since “resilience” as an outcome is modifiable and assessable (i.e., how well did you bounce back from the stressful event?) and outcome-oriented definitions and assessments require a baseline of stress, Black women are suitable candidates for resilience-based interventions, because they have dealt with a history of injustice [20,31,33,34]. Thus, our goal is to evaluate the relationship as a foundational step to understanding whether assessing resilience could be beneficial in lifestyle interventions for older women, and to test whether that relationship differs by race/ethnicity—especially among Black women.

## 2. Materials and Methods

### 2.1. Study Design

Since 1993, the Women’s Health Initiative (WHI) has investigated strategies to prevent and control the common causes of morbidity and mortality, including cancer, fractures, and cardiovascular events among older American women. The longitudinal study has been conducted at 40 research institutions across the United States and has been extended three times—the WHI Extension Study I (2005–2010), Extension Study II (2010–2015), and Extension Study III (2015–2027). Details about the WHI study, as well as the first and second extension studies’ rationales, designs, and consent processes, have been previously reported [24,25]. Participants in the original WHI clinical trial and observational study enrolled at baseline (between 1993 and 1998) and were followed until 2005. In 2005, 2010, and 2015, participants from the original study were given the opportunity to enroll in extension studies for follow up.

The WHI originally enrolled 161,808 women aged 50–79 between 1993 and 1998. Of the 161,808 women who have participated in the WHI, we considered all women enrolled in the WHI Extension Study II who had available data on resilience collected in 2011 (*N* = 78,525) in our cohort who were then aged 62–85+. We excluded women who identified as American Indian, Alaska Native, or those categorized as ‘other’ due to their low numbers (*N* = 1133; 1%), consistent with an aim of this study to evaluate whether race/ethnicity is an effect modifier with adequate power. This yielded a final analytic cohort of *N* = 77,395. See Figure S1 for an illustration of the study dropout rate and missing data. Written, informed consent was obtained from all study participants at enrollment, and as part of each extension study.

As noted above, our objective was to examine the relationship between self-reported resilience and the following 5 CVD-protective health behaviors: (1) diet quality (top 2 quintiles of the Healthy Eating Index 2015 vs. bottom 3); (2) smoking status (current non-smoker vs. smoker), (3) physical activity (adequate (≥ 150 min of total (walking, moderate, and vigorous) physical activity per week) vs. inadequate (< 150 min]), (4) meeting the recommended hours of sleep per night (7–9 h) vs. more or less than recommended; and (5) consuming alcohol moderately (consuming alcoholic drink(s) 1–7 days per week). Each behavior was assessed against the backdrop of recommendations for preventing CVD [2].

In the absence of a national guideline for diet quality, we used epidemiological evidence from other large observational cohorts, such as the Multiethnic Cohort study [35]. The main study measures were collected in 2011, with the exception of diet quality, which was collected at baseline (between 1993 and 1998). All study measures were collected by mail via multiple surveys to accommodate a large sample size and maintain participation over time.

### 2.2. Measures

#### 2.2.1. CVD-protective Health Behavior Outcomes

Better diet quality: Drawing on the validated WHI food frequency questionnaire collected at baseline [36], diet quality was measured using the Healthy Eating Index 2015 (HEI-2015). This includes 13 dietary components (9 adequacy and 4 moderation) that sum to a total of 100 points. Higher scores indicate a higher diet quality. We decided to use the top 2 quintiles of the HEI-2015 based on recent evidence demonstrating that diet quality scores that fall into these upper-level distributions are associated with a significantly lower risk of CVD-related and all-cause mortality in women [35,37,38]. In terms of the data collected, higher scores reflected adherence to the 2015 Dietary Guidelines for Americans and a superior diet quality.

Non-smoking status: Even if a person has a history of smoking, being a current non-smoker is a protective behavior against CVD [39]. Accordingly, participants were asked to answer “yes” or “no” to the following question: “Do you smoke cigarettes now?” We did not measure lifetime smoke exposure.

Recommended physical activity: Total min of recreational physical activity—including mild, moderate, and strenuous physical activity (per week)—was derived from questions about the participants’ routine physical activities and exercise regimens. Participants were asked about how often and how long they engaged in various activities, such as walking, dancing, biking outdoors, swimming, and tennis. Using the 2019 American Heart Association’s physical activity recommendation as a guide, we classified whether participants achieved ≥ 150 min of total physical activity per week [40], noting that even light-intensity exercise provides significant health benefits in older women [41,42].

Adequate sleep: Participants were asked “About how many hours of sleep did you get on a typical night during the past 4 weeks?” We classified responses based on the recommended amount of 7–9 h [43]. Of note, Sands-Lincoln and colleagues (2013) examined both shorter (≤ 5 h) and longer (≥ 10 h) sleep periods in the context of incidence of CVD among postmenopausal women in the WHI [44]. They found that women who self-reported both shorter and longer sleep duration demonstrated significantly higher rates of coronary heart disease (25%) and CVD (19%) in age- and race-adjusted models [44].

Moderate alcohol consumption: Participants were asked, “In the past 3 months, how often have you had drinks containing alcohol?” Their response items ranged from “0 = Never,” “1 = 1 or 2 times per week,” to “7 = every day.” Based on these responses and the assumption that women did not consume drinks more frequently than 1 drink each day, we developed a binary variable for moderate alcohol use to describe participants who reported consuming alcoholic drink(s) 1–7 days per week (moderate drinkers) [45,46,47].

#### 2.2.2. Exposures and Covariates

Resilience: Resilience was measured using an abbreviated version of the brief resilience scale (BRS) [20,48]. The original BRS was tested in *N* = 354 individuals who were subdivided into 4 groups, including 2 behavioral medicine samples (*N* = 112 cardiac patients and *N* = 50 older women with fibromyalgia). In previous studies, the BRS proved to be a key indicator of aging well in the WHI cohort [49]. Similarly, the scores were moderately high in our population. Because BRS distribution was skewed toward higher resilience, total scores were categorized into low (0.1–2.9), medium (3.0–4.2), and high (4.3–5.0) resilience levels based on the authors’ suggested cut-offs to examine possible non-linear associations [48]. Using a 5-point Likert scale (strongly disagree/agree), our participants rated the following statements: “I tend to bounce back quickly after hard times,” “It does not take me long to recover from a stressful event,” and “I have a hard time making it through stressful events.” Cronbach’s alpha coefficient for the 3 items was 0.74, suggesting relatively high internal consistency.

Stressful life events: Stressful life events were assessed through 12 questions drawn from a modified life change measure from the Beta-Blocker Heart Attack Trial [50]. Specifically, our study participants were asked whether they had experienced any of 12 different stressful life events in the past year, and, if so, how much each one had upset them. For example, they were asked: “Did you have any major problems with money?” Participant response options included “No”, “Yes, and it upset me: Not too much,” “Yes, and it upset me: Moderately,” and “Yes, and it upset me: Very much.” Each response was scored 0–3 per question, and all 12 items were summed such that totals scores ranged from 0 to 36. A higher score indicated that participants experienced an increased number of stressful life events that were very upsetting to them.

Additional variables: The sociodemographic variables that were assessed included self-reported age (in years); race/ethnicity (Black or African American (Black), non-Hispanic white (not Hispanic origin), Hispanic or Latina (Latina), Asian or Pacific Islander); education level (< high school, high school or general education diploma, some college, 4 year college degree or more); annual household income (<$20,000 per year, $20,000–$34,999, $35,000–$49,999, $50,000–$74,999, $75,000+; and marital status (never married, divorced or separated, widowed, presently married/married-like relationship). Other CVD risk factors considered were body mass index (BMI), and presence of diabetes, hypertension, and high cholesterol. Note, BMI was calculated from the weight and height measurements taken at baseline (1993–1998). The presence of diabetes, hypertension, and high cholesterol was determined through self-reported diagnosis or treatment of these conditions. For diabetes, self-reported treatment included medication, insulin, and diet or exercise. Additionally, the depression scale was computed based on the short form of the Center for Epidemiologic Studies Depression Scale (CES-D), and values ranged from 0 to 1, with a higher score indicative of greater likelihood of depression [51,52,53]. Note, authors of the CES-D consider those with a score greater or equal to 0.06 as depressive.

### 2.3. Statistical Analysis

Descriptive statistics were used to describe stressful life events (SLEs), sociodemographic information, and CVD-protective behaviors by level of resilience.

To investigate the association between resilience and CVD-protective behaviors, we fit a series of multivariable logistic regression models, adjusted for sequentially-added variables. The covariates included in all models were pre-specified. Initially, we used logistic regression analysis to: fit unadjusted models (Model 0) and models adjusted for the stressful life event score (Model 1); the stressful life event score and sociodemographic information (age, race/ethnicity, education) (Model 2); and the stressful life event score, sociodemographic, and additional CVD risk factors (BMI, hypertension, diabetes, and/or high cholesterol) (Model 3). To assess whether the association differed among racial/ethnic groups, we added a statistical interaction term between resilience and race/ethnicity to the fully adjusted models (Model 4). Black women with low resilience were selected as the reference group because they were at the highest risk of CVD and poor health behaviors [2,10]. Resilience (3 levels) and race/ethnicity (4 levels) were both treated as categorical variables, yielding 11 interaction terms. ANOVA was used to jointly test the null hypothesis that the coefficient on the statistical interaction terms were equal to 0. Overall, 5 models were fit for each of the 5 outcomes, totaling 25 models. As a post-hoc sensitivity analysis, we included depression scale as an additional covariate to the fully-adjusted model and the fully-adjusted model with an interaction term between resilience and race/ethnicity.

To address missing data, we created 5 datasets using multiple imputations by chained equations (MICE) that were then combined using Rubin’s rules. Analyses were performed in Stata 13.1 (StataCorp. 2013. *Stata Statistical Software: Release 13*. College Station, TX, USA: StataCorp LP) and SAS 9.4 (SAS Institute, Inc., Cary, NC, USA). All tests were 2-sided and performed at the 0.05 significance level. Two authors (H.H. and F.Q.) had full access to all of the WHI data required for this study and take responsibility for its integrity and the data analysis.

## 3. Results

Of the 77,395 WHI Extension Study II participants, most women self-reported medium (36.8%) or high (35.7%) levels of resilience. Few women reported low resilience levels (5.6%) (for more on this, see Table 1). Women who self-reported high resilience levels tended to have lower stressful life events and higher socioeconomic resources compared to women with low or medium levels of resilience. On average, women who self-reported high levels of resilience experienced fewer stressful life events ((mean ± standard deviation) 2.67 ± 2.72 vs. 4.14 ± 3.80); had higher levels of education (49.8% had a 4 year college degree or more vs. 40.9%); and had higher annual household incomes (27.4% reported $75,000 vs. 18.6%), compared to women who reported low resilience. The racial/ethnic distribution among women in the high-resilience group was consistent with that of the total sample, with Black women being slightly over-represented.

**Figure 1:**
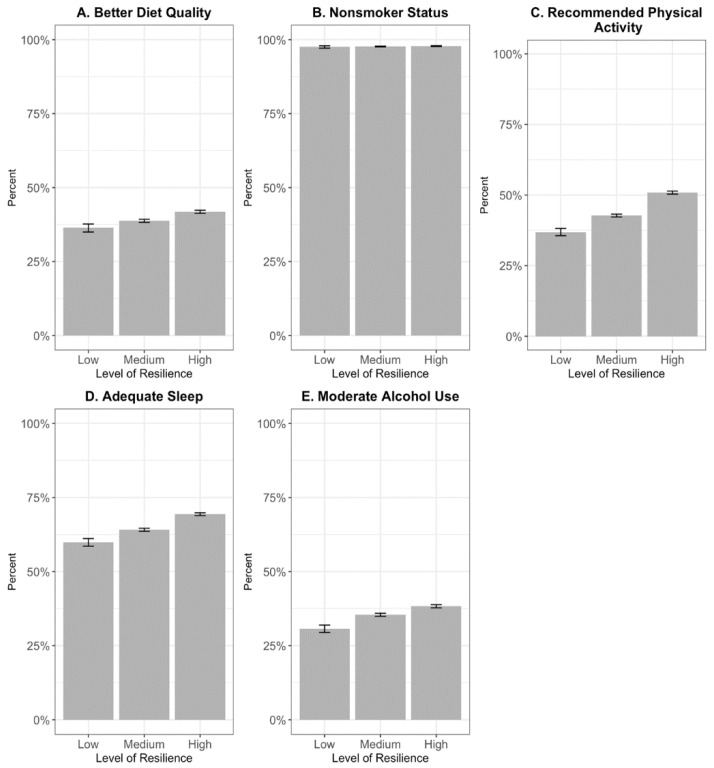
Percent of women achieving positive CVD-protective health behaviors. The bars represent 95% confidence intervals for the percentages. The letters represent the following: A. Better Diet Quality: Top 2 quintiles of the Healthy Eating Index 2015 (HEI-2015); B. Non-Smoker Status: current non-smoker; C. Recommended Physical Activity: 150 min of recreational physical activity per week; D. Adequate Sleep: sleeping 7–9 h per night; and E. Moderate Alcohol Use: consuming alcoholic drink(s) 1–7 days per week.

In terms of CVD risk factors, women with high resilience showed a lower prevalence of obesity (26.3% vs. 27.2%); diabetes (13.2% vs. 15.5%); hypertension (55.1% vs. 62.1%); and high cholesterol (27.4% vs. 29.6%) compared to women with low resilience. Women with high resilience also had the highest engagement in protective health behaviors according to CVD prevention guidelines (see Table 2 and Figure 1).

Compared to those who reported low resilience in the full model (Model 3), given similar stressful life events, sociodemographic factors (i.e., age, race/ethnicity and education), and CVD risk factors (i.e., BMI, hypertension, diabetes, and/or high cholesterol), women who reported high resilience had significantly greater odds of diet quality scores in the top 2 quintiles (OR = 1.22; CI:1.15–1.30); engaging in 150 min of recreational physical activity per week (OR = 1.56; CI:1.47–1.66); sleeping the recommended 7–9 h (OR = 1.36; CI:1.28–1.44); and moderately consuming alcohol (OR = 1.28; CI:1.20–1.37).

Similarly, compared to those who reported low resilience, women who reported medium resilience had significantly greater odds of diet quality scores in the top quintiles (OR = 1.09; CI:1.02–1.16); engaging in 150 min of recreational physical activity per week (OR = 1.23; CI:1.15–1.31); sleeping the recommended 7–9 h (OR = 1.14; CI:1.07–1.21); and moderately consuming alcohol (OR = 1.20; CI:1.13–1.28), after adjusting for covariates. Neither high (OR = 1.02; CI: 0.85–1.24) nor medium (OR = 0.99; CI: 1.02–1.16) resilience levels were significantly associated with non-smoking status (see Table 3).

Additionally, women who reported higher levels of resilience overall had lower likelihood of depression ((mean ± standard deviation) 0.0066 ± 0.040 vs. 0.036 ± 0.12 vs. 0.11 ± 0.22 for high, medium, and low resilience, respectively). In our post-hoc sensitivity analysis, adding depression to the fully adjusted model and interaction model did not substantially alter our findings (see Tables S2 and S3).

**Figure 2:**
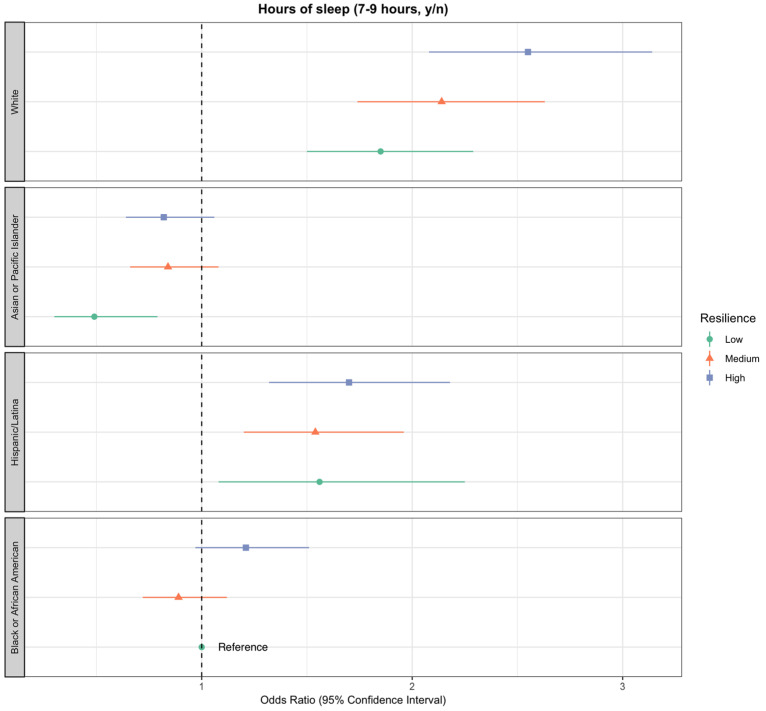
Estimated associations between self-reported psychological resilience and adequate sleep (recommended 7–9 h per night), adjusted for stressful life events (SLEs), sociodemographic information (age, race/ethnicity, and education), additional CVD-protective risk factors (BMI, diabetes, hypertension, and high cholesterol), and interaction between race/ethnicity and resilience. The odds ratio for each category is displayed along with bars representing 95% confidence intervals.

These associations differed by racial/ethnic groups but only for recommended h of sleep (*p* for interaction = 0.03; see Figure 2 and Table S1). All racial/ethnic groups who reported high resilience demonstrated the increased odds of sleeping the recommended 7–9 h compared to those with medium and low resilience, with the exception of Asian and Pacific Islanders. Compared to Black women who reported low resilience in the full model, non-Hispanic white women who reported high resilience had the greatest odds of achieving recommended sleep hours (OR = 2.55; CI: 2.08–3.14), followed by Latinas (OR = 1.70; CI: 1.32–2.18) and Black women (OR = 1.21; CI: 0.97–1.51). Non-Hispanic white women were the only group where a decrease in the odds of achieving recommended sleep hours (7–9) was seen for each corresponding decrease in resilience levels (OR = 1.68; CI: 0.99–2.85 for high, OR = 1.66; CI: 0.98–2.80 for medium, and OR = 1.57; CI: 0.90–2.75 for low vs. Black women with low resilience). Note, we did not observe effect modification between race/ethnicity and resilience for the diet quality, alcohol intake, and non-smoker status outcomes (see Figures S2–S5).

## 4. Discussion

Our findings are consistent with the body of evidence that suggests a high level of self-reported resilience is positively associated with engaging in protective health behaviors [10,22,23,24,25,26,27]. Granted, there is no consensus on the definition of “resilience” or a gold-standard measurement [54] used in related studies. Moreover, the fact that researchers have used different resilience scales across studies limited our ability to make direct comparisons. Accordingly, we contextualized our findings by comparison to national averages, clinically significant cut-points, and previous studies focused on self-reported resilience and CVD-protective behaviors.

Overall, we found that women who reported high levels of resilience had 22% greater odds of reporting HEI-2015 diet quality scores in the top 2 quintiles of our study sample (Q1:74.7–96.2; Q2:69.0–74.7), after adjusting for covariates. Among women in the high-resilience group, the average HEI-2015 score was 66.1 (±10.2), which is consistent with the national average for older adults aged 65+, and has been associated with lower risk of mortality from all-cause CVD and cancer in racially and ethnically-diverse older women [35]. Considering the importance of improving and maintaining high diet quality in aging populations and racial/ethnic minorities, further research needs to be conducted to identify other resources that can foster resilience in older women [55,56].

Participants who reported high levels of resilience had 56% greater odds of meeting the national physical activity guidelines (of at least 150 min per week) for chronic disease prevention, compared to women with medium and low levels of resilience. This is consistent with Heberg and colleagues’ findings [25]. We also observed a positive relationship between resilience and physical activity in women who had experienced similar amounts of stressful life events. Hence, future studies should examine the biological pathways whereby physical fitness might confer resilience, including optimizing neuroendocrine and physiological responses to psychosocial stressors [57,58]. Further research should also be conducted on the role of resilience in boosting physical fitness, in response to different types of stressors that may affect older women from diverse backgrounds.

With respect to sleep, we found that women who reported high levels of resilience had 36% greater odds of getting the recommended 7–9 h per night, after adjusting for covariates. Our findings add to the evidence that suggests there is an overall positive association between high resilience and healthy sleep behaviors [27,59,60]. Notably, sleep duration was the only CVD-protective behavior whose relationship with resilience was moderated by racial/ethnic group; the association seems to be particularly strong for non-Hispanic whites.

Considering the increasing prevalence of poor sleep quality among American adults, there is a critical need for more studies investigating the link between higher resilience and healthy sleep behaviors [61]. This is highly pertinent to public health policies and educational initiatives for vulnerable populations, given substantial evidence linking unhealthy sleeping patterns to racial/ethnic discrimination [62]. On that note, researchers should consider the reciprocal relationships between sleep, physical activity, and dietary behaviors to understand the role of resilience and multiple unhealthy behaviors [63,64].

Compared to those in the low and high resilience categories, Black women who reported medium resilience had the lowest odds of getting the recommended 7–9 h of sleep per night. Where this U-shaped relationship partially contradicted our hypothesis, it is perhaps best understood by taking into account the phenomenon known as the “Superwoman” role. Specifically, Black women may feel pressured by their community as well as the larger society to fulfill a legacy of strength, i.e., pulling oneself up by the bootstraps to function in professional and familial roles even in the face of excessive chronic and daily stressors, presumably stemming from racism and sexism [34,65,66,67,68]. While maintaining the “Superwoman” identity has been viewed as an asset that has contributed to the resilience of Black women, it has also been linked to decreased emotional support, increased psychological stress and depressive symptoms as well as poor health behaviors, including poor sleep quality and sacrificing sleep in order to reach one’s goals [65,67,69]. Unlike the other CVD-protective behaviors, among Black Americans, sleeping is stigmatized as unproductive and is associated with not being a hard worker [70]. It stands to reason that integrating formal resilience-building skills (i.e., how to access and mobilize resources) into lifestyle interventions that address the factors that compete with healthy sleep behaviors may be especially important in promoting positive health outcomes among Black women.

Given the relatively small number of women in our sample who reported drinking alcohol and smoking, we cannot offer conclusive statements about their relationship to resilience. Briefly, 36.4% (*n* = 27,762) of our sample reported being moderate drinkers, consuming alcoholic drink(s) 1–7 days per week. Women in the high-resilience group had 37% greater odds of being a moderate drinker versus not drinking, possibly adhering to evidence-based recommendations for moderate alcohol use (1 alcoholic drink per day) [71]. While resilience was not significantly associated with being a non-smoker in our study, previous studies—with a comparable sample of non-smokers and smokers—reported a significant association [23,24]. Thus, more research is needed to evaluate the relationship between resilience, moderate alcohol consumption, and being a non-smoker in older women.

Even though our study helps to understand the relationship between self-reported resilience and CVD-protective behaviors among aging American women, it is not without limitations. Our findings may only be generalizable to older women, the mean age of our sample 77 years. Tied to this, the women who consented to remain in the Extension Study II may be healthier and/or more driven to stay in the study than the general population who dropped out or declined enrollment. What’s more, due to its cross-sectional nature, we cannot establish causality between BRS resilience and CVD-protective behaviors. Resilience could influence healthy behaviors, but healthy behaviors could also foster resilience. To confirm the directionality of the association, we would need to conduct a longitudinal analysis.

Further, due to the relatively small number of women of color, relative to non-Hispanic white participants, the confidence intervals for estimates in the low-resilience group are quite wide. This suggests that the corresponding estimates must be interpreted with care—particularly for Asian or Pacific Islander women. Further, since all measures self-reported at single timepoints, they are potentially affected by reporting bias and issues of reliability. Measurement of never smoking, as opposed to current smoking, may have increased the likelihood of our detecting a signal between smoking and resilience. We add this limitation to lines. Although most independent and dependent variables were collected at the same time (in 2011), a few were collected at baseline (1993–1998), including the WHI food frequency questionnaire. The time lag between the dietary assessment and resilience measure may have attenuated the relationship.

Although the cut-offs for our binary outcomes followed evidence-based prevention guidelines, this may have limited our ability to capture specific recommendations. For instance, our measure of alcohol intake is based on weekly frequency, yet the evidence-based recommendation relied on a quantitative estimation of alcohol consumed. Our measure assumes that the women in our sample did not consume more than one drink, 14 g of alcohol during the 1–7 times they reported drinking. This assumption was based on the high proportion of women who reported having had less than 1 drink per week and on previous epidemiological evidence suggesting that a large proportion of the WHI abstain or are light drinkers, with few heavy drinkers [72]. Lastly, our primary measure of resilience was limited by the shortened version of the BRS for assessing the ability to bounce back.

Nonetheless, our findings are significant. To the best of our knowledge, this is the first study to evaluate self-reported resilience, several CVD-protective health behaviors, and racial/ethnic interactions in a large sample of racially and ethnically-diverse older American women. We also used a rigorous dietary assessment and a validated food frequency questionnaire-based comprehensive diet quality score, from which the latest index was calculated (HEI-2015) [36].

Overall, this study draws attention to the potential of resilience, a modifiable and assessable variable [73], to inform behavioral interventions focused on prevention and health promotion in ethnically and racially diverse communities of older American women. Tied to this, because the BRS takes an outcome-oriented approach that defines resilience in terms of how well someone bounces back from stress, it is ideal for interventions.

## 5. Conclusions

High levels of perceived resilience are significantly associated with engaging in CVD-protective behaviors. This association holds across different racial/ethnic groups. Further research investigating whether and how resilience can moderate the relationship between stressors, including perceived discrimination and CVD-protective behaviors, is warranted, as this study is an observational cross-sectional study. Moreover, it is worthwhile to investigate the efficacy of resilience-based interventions to promote adherence to CVD prevention guidelines and conduct longitudinal follow up in future randomized trials. Taken together, this study serves as a foundational step in understanding resilience as a factor that can be used to inform strength-based approaches to health promotion, particularly in vulnerable populations such as Black women, who are able to remain resilient in the face of historical injustices and contemporary discrimination.

## Figures and Tables

**Table 1:** Description of perceived stress, sociodemographic information, and other health factors by low, medium, and high levels of self-reported psychological resilience from participants in the Women’s Health Initiative (WHI) Extension Study II.

Characteristics	Total N (%) *N* = 77,395	Low (BRS 1.0–2.9) *N* = 5496	Medium (BRS 3.0–4.2) *N* = 36,638	High (BRS 4.3–5.0) *N* = 35,261	Absolute Standardized Difference (ASD)
Stressful life events, (mean (sd)), range: 0–36	3.20 (3.13)	4.14 (3.80)	3.57 (3.31)	2.67 (2.72)	0.301
Missing	287	45	140	102	
Sociodemographic information					
Age in years at collection of resilience, (mean (sd))	77.0 (6.4)	77.7 (6.7)	77.4 (6.4)	76.5 (6.3)	0.122
Age categories at collection of resilience (%):					
62–74	28,356 (36.6)	1846 (33.6)	12,503 (34.1)	14,007 (39.7)	
75–84	37,367 (48.3)	2608 (47.5)	18,119 (49.5)	16,640 (47.2)	
85+	11,672 (15.1)	1042 (19.0))	6016 (16.4)	4614 (13.1)	
Missing	0	0	0	0	
Race/Ethnicity (%):					0.070
Black or African American	4475 (5.8)	387 (7.0)	1914 (5.2)	2174 (6.2)	
Non-Hispanic white (not of Hispanic origin)	69,448 (89.7)	4840 (88.1)	33,024 (90.1)	31,584 (89.6)	
Hispanic/Latina	1891 (2.4)	177 (3.2)	895 (2.4)	819 (2.3)	
Asian or Pacific Islander	1581 (2.0)	92 (1.7)	805 (2.2)	684 (1.9)	
Missing	0	0	0	0	
Education level (%):					0.152
≤High School	1930 (2.5)	228 (4.2)	1037 (2.8)	665 (1.9)	
High School or General Education Degree	11,573 (15.1)	943 (17.3)	6077 (16.7)	4553 (13.0)	
Some college	27,716 (36.0)	2052 (37.7)	13,290 (36.5)	12,374 (35.3)	
≥College degree	35,674 (46.4)	2226 (40.9)	16,011 (44.0)	17,437 (49.8)	
Missing	502	47	223	232	
Annual family income (%):					0.194
<20,000	7305 (10.0)	735 (14.2)	3839 (11.1)	2731 (8.1)	
20,000–34,999	15,742 (21.4)	1278 (24.6)	7896 (22.8)	6568 (19.6)	
35,000–49,999	15,650 (21.3)	1123 (21.7)	7569 (21.8)	6958 (20.7)	
50,000–74,999	16,955 (23.1)	1085 (20.9)	7761 (22.4)	8109 (24.2)	
75,000+	17,761 (24.2)	967 (18.6)	7601 (21.9)	9193 (27.4)	
Missing	3982	308	1972	1702	
Marital status (%):					0.045
Never married	3142 (4.1)	246 (4.5)	1472 (4.0)	1424 (4.1)	
Divorced or separated	11,386 (14.8)	855 (15.6)	5137 (14.1)	5394 (15.3)	
Widowed	9824 (12.7)	737 (13.5)	4699 (12.9)	4388 (12.5)	
Presently married/married-like relationship	52,781 (68.4)	3639 (66.4)	25,206 (69.0)	23,936 (68.1)	
Missing	262	19	124	119	
Baseline health status					
BMI, (mean (sd))	27.5 (5.6)	27.6 (5.8)	27.5 (5.7)	27.4 (5.6)	0.019
Missing	658	45	309	304	
Diet quality (HEI-2015) (mean (sd)), range: 0–100	65.7 (10.2)	64.8 (10.5)	65.4 (10.2)	66.1 (10.2)	0.088
Missing	136	11	64	61	
CVD-risk factors					
Obese, (BMI kg, m^2^ 30+) (%):					
Yes	20,286 (26.4)	1483 (27.2)	9625 (26.5)	9178 (26.3)	0.014
No	56,451 (73.6)	3968 (72.8)	26,704 (73.5)	25,779 (73.7)	
Missing	658	45	309	304	
Diabetes (%):					0.045
Yes	10,793(13.9)	852 (15.5)	5304 (14.5)	4637 (13.2)	
No	66,602 (86.1)	4644 (84.5)	31,334 (85.5)	30,624 (86.8)	
Hypertension (%):					0.094
Yes	44,472 (57.5)	3413 (62.1)	21,619 (59.0)	19,440 (55.1)	
No	32,923 (42.5)	2083 (37.9)	15,019 (41.0)	15,821 (44.9)	
High cholesterol (%):					0.077
Yes	20,385 (26.3)	1626 (29.6)	10,138 (27.7)	8621 (24.4)	
No	57,010 (73.7)	3870 (70.4)	26,500 (72.3)	26,640 (75.6)	

**Table 2:** CVD-protective health behaviors by low, medium, and high levels of self-reported psychological resilience from participants in the Women’s Health Initiative (WHI) Extension Study II.

CVD-Protective Health Behaviors	Total *N* (%) *N* = 77,395	Low (BRS 1.0–2.9) *N* = 5496	Medium (BRS 3.0–4.2) *N* = 36,638	High (BRS 4.3–5.0) *N* = 35,261
Better diet quality (HEI-2015) top 2 quintiles (Q1:74.7–96.2; Q2:69.0–74.7) (%):				
Yes	30,904 (40.0)	1996 (36.4)	14,180 (38.8)	14,728 (41.8)
No	46,355 (60.0)	3489 (63.6)	22,394 (61.2)	20,472 (58.2)
Missing	136	11	64	61
Non-smoker (%):				
Yes	74,514 (97.7)	5246 (97.5)	35,242 (97.7)	34,026 (97.8)
No	1739 (2.3)	133 (2.5)	842 (2.3)	764 (2.2)
Missing	1142	117	554	471
Min of recreational physical activity per week (mean (sd)):	166.5 (177.1)	133.4 (163.2)	151.4 (165.5)	187.3 (188.0)
Missing	1136	108	590	438
Recommended 150 min of recreational physical activity per week (%):				
Yes	35,099 (46.0)	1987 (36.9)	15,407 (42.7)	17,705 (50.8)
No	41,160 (54.0)	3401 (63.1)	20,641 (57.3)	17,118 (49.2)
Missing	1136	108	590	438
Sleep duration, h per night (%):				
≤5	6484 (8.4)	656 (12.0)	3423 (9.4)	2405 (6.9)
6	18,616 (24.2)	1397 (25.6)	9177 (25.2)	8042 (22.9)
7	26,748 (34.8)	1630 (29.9)	12,373 (34.0)	12,745 (36.3)
8	18,610 (24.2)	1152 (21.1)	8332 (22.9)	9126 (26.0)
9	5555 (7.2)	483 (8.9)	2616 (7.2)	2456 (7.0)
10+	914 (1.2)	137 (2.5)	484 (1.3)	293 (0.8)
	468	41	233	194
Recommended 7–9 h of sleep per night during past 4 months (%):				
Yes	50,913 (66.2)	3265 (59.9)	23,321 (64.1)	24,327 (69.4)
No	26,014 (33.8)	2190 (40.1)	13,084 (35.9)	10,740 (30.6)
Missing	468	41	233	194
Alcohol use in past 3 months (%):				
Never/less than 1 time per week	48,486 (63.6)	3723 (69.3)	23,303 (64.6)	21,460 (61.7)
1 to 4 times per week	15,305 (20.1)	953 (17.7)	7015 (19.4)	7337 (21.1)
5 to 6 times per week	5809 (7.6)	299 (5.6)	2710 (7.5)	2800 (8.1)
7 times per week (everyday)	6648 (8.7)	396 (7.4)	3049 (8.5)	3203 (9.2)
Missing	1147	125	561	461
Moderate alcohol use Had alcoholic drink(s) 1–7 time(s) per week (%):				
Yes	27,762 (36.4)	1648(30.6)	12,774 (35.4)	13,340 (38.3)
No	48,486 (63.6)	3723 (69.3)	23,303 (64.6)	21,460 (61.7)
Missing	1147	125	561	461

**Table 3:** Estimated associations between self-reported psychological resilience and CVD-protective health behaviors, adjusted for stressful life events (SLEs), sociodemographic information (age, race/ethnicity, and education), and additional CVD-protective risk factors (BMI, diabetes, hypertension, and dyslipidemia). Low BRS was designated as the reference group for all models. Each cell contains the estimated odds ratio (95% confidence interval).

Outcome	Unadjusted	Model 1	Model 2	Model 3
		SLEs	SLEs Sociodemographic	SLEs Sociodemographic Additional CVD risk factors
Better diet quality (top 2 quintiles of HEI-2015, yes/no)	BRS medium: 1.11 (1.04, 1.17) BRS high: 1.26 (1.19, 1.33)	BRS medium: 1.09 (1.02, 1.15) BRS high: 1.20 (1.13, 1.27)	BRS medium: 1.08 (1.02, 1.15) BRS high: 1.20 (1.13, 1.28)	BRS medium: 1.08 (1.02, 1.15) BRS high: 1.22 (1.14, 1.29)
Non-smoker (yes/no)	BRS medium: 1.06 (0.88, 1.28) BRS high: 1.13 (0.94, 1.36)	BRS Medium: 1.01 (0.84, 1.22) BRS high: 1.01 (0.84, 1.22)	BRS Medium: 1.00 (0.83, 1.20) BRS high: 1.03 (0.86, 1.25)	BRS medium: 0.99 (0.82, 1.20) BRS high: 1.02 (0.85, 1.24)
Recommended Physical Activity (≥150 min per week, yes/no)	BRS medium: 1.28 (1.21, 1.36) BRS high: 1.78 (1.68, 1.89)	BRS medium: 1.26 (1.19, 1.34) BRS high: 1.70 (1.60, 1.80)	BRS medium: 1.22 (1.15, 1.29) BRS high: 1.54 (1.45, 1.63)	BRS medium: 1.23 (1.15, 1.31) BRS high: 1.56 (1.47, 1.66)
Adequate Sleep (7–9 h of sleep per night, yes/no)	BRS medium: 1.20 (1.13, 1.27) BRS high: 1.52 (1.44, 1.61)	BRS medium: 1.16 (1.10, 1.23) BRS high: 1.42 (1.34, 1.50)	BRS medium: 1.14 (1.07, 1.21) BRS high: 1.36 (1.28, 1.44)	BRS medium: 1.14 (1.07, 1.21) BRS high: 1.36 (1.28, 1.44)
Moderate Alcohol Use (had alcoholic drink(s) 1–7 time(s) per week, yes/no)	BRS medium: 1.24 (1.17, 1.32) BRS high: 1.41 (1.32, 1.50)	BRS medium: 1.23 (1.16, 1.31) BRS high: 1.37 (1.29, 1.46)	BRS medium: 1.19 (1.12, 1.27) BRS high: 1.26 (1.18, 1.35)	BRS medium: 1.20 (1.13, 1.28) BRS high: 1.28 (1.20, 1.37)

Self-reported psychological resilience (resilience) was measured by a shortened 3-item version of the brief resilience scale (BRS).

## Supplementary Materials

The following are available online at https://www.mdpi.com/2072-6643/12/7/2107/s1, Figure S1: Study flowchart illustrating participant dropout, Figure S2: Estimated associations between self-reported psychological resilience and moderate alcohol use, Figure S3: Estimated associations between self-reported psychological resilience and better diet quality, Figure S4: Estimated associations between self-reported psychological resilience and being current non-smoker, Figure S5: Estimated associations between self-reported psychological resilience and recommended physical activity, Table S1: Results from full model including interaction between race/ethnicity and resilience, Table S2: Sensitivity analysis: estimated associations between self-reported psychological resilience and CVD-protective health behaviors, and Table S3. Sensitivity analysis: results from full model including interaction between race/ethnicity and resilience.

## References

[1] Garcia M., Mulvagh S.L., Merz C.N.B., Buring J.E., Mason J.E. (2016). Cardiovascular disease in women: Clinical perspectives. Circ. Res..

[2] Mozaffarian D., Benjamin E.J., Go A.S., Arnett D.K., Blaha M.J., Cushman M., Das S.R., De Ferranti S., Després J.P., Fullerton H.J. (2016). Executive summary: Heart disease and stroke statistics—2016 update: A report from the American Heart Association. Circulation.

[3] Krittanawong C., Tunhasiriwet A., Wang Z., Zhang H., Prokop L.J., Chirapongsathorn S., Aydar M., Sun T., Kitai T. (2017). Association between short and long sleep duration and cardiovascular outcomes? A systematic review and meta-analysis. J. Am. Coll. Cardiol..

[4] Ramachandran H.J., Wu V.X., Kowitlawakul Y., Wang W. (2016). Awareness, knowledge and healthy lifestyle behaviors related to coronary heart disease among women: An integrative review. Heart Lung.

[5] Mosca L., Mochari H., Christian A., Berra K., Taubert K., Mills T., Burdick K.A., Simpson S.L. (2006). National study of women’s awareness, preventive action, and barriers to cardiovascular health. Circulation.

[6] Shaver J.L., Johnston S.K., Lentz M.J., Landis C.A. (2002). Stress exposure, psychological distress, and physiological stress activation in midlife women with insomnia. Psychosom. Med..

[7] Dimsdale J.E. (2008). Psychological stress and cardiovascular disease. J. Am. Coll. Cardiol..

[8] Geiker N.R.W., Astrup A., Hjorth M.F., Sjödin A., Pijls L., Markus C.R. (2018). Does stress influence sleep patterns, food intake, weight gain, abdominal obesity and weight loss interventions and vice versa?. Obes. Rev..

[9] Seib C., Whiteside E., Lee K., Humphreys J., Tran T.H.D., Chopin L., Anderson D. (2014). Stress, lifestyle, and quality of life in midlife and older Australian women: Results from the stress and the health of women study. Women Health Issues.

[10] Felix A.S., Lehman A., Nolan T.S., Sealy-Jefferson S., Breathett K., Hood D.B., Addison D., Anderson C.M., Cené C.W., Warren B.J. (2019). Stress, resilience, and cardiovascular disease risk among black women: Results from the women’s health initiative. Circ. Cardiovasc. Qual. Outcomes.

[11] Albert M.A., Durazo E.M., Slopen N., Zaslavsky A.M., Buring J.E., Silva T., Chasman D., Williams D.R. (2017). Cumulative psychological stress and cardiovascular disease risk in middle aged and older women: Rationale, design, and baseline characteristics. Am. Heart J..

[12] Williams D.R., Lawrence J.A., Davis B.A. (2019). Racism and health: Evidence and needed research. Annu. Rev. Public Health.

[13] Gee C.G., Ford C.L. (2011). Structural racism and health inequities: Old issues, new directions. Du Bois Rev..

[14] Woods-Giscombé C.L., Lobel M. (2008). Race and gender matter: A multidimensional approach to conceptualizing and measuring stress in African American women. Cult. Divers. Ethn. Minority Psychol..

[15] Williams D.R., Collins C. (2001). Racial residential segregation: A fundamental cause of racial disparities in health. Public Health Rep..

[16] Lewis J.A., Williams M.G., Peppers E.J., Gadson C.A. (2017). Applying intersectionality to explore the relations between gendered racism and health among Black women. J. Couns. Psychol..

[17] Odoms-Young A.M. (2018). Examining the impact of structural racism on food insecurity: Implications for addressing racial/ethnic disparities. Fam. Community Health.

[18] Wen M., Zhang X., Harris C.D., Holt J.B., Croft J.B. (2013). 2013 Spatial disparities in the distribution of parks and green spaces in the USA. Ann. Behav. Med..

[19] Black L.L., Johnson R., Van Hoose L. (2015). The relationship between perceived racism/discrimination and health among black American women: A review of the literature from 2003 to 2013. J. Racial Ethn. Health Disparities.

[20] Smith B.W., Dalen J., Wiggins K., Tooley E., Christopher P., Bernard J. (2008). The brief resilience scale: Assessing the ability to bounce back. Int. J. Behav. Med..

[21] MacLeod S., Musich S., Hawkins K., Alsgaard K., Wicker E.R. (2016). The impact of resilience among older adults. Geriatr. Nurs..

[22] Lutz L.J., Gaffney-Stomberg E., Williams K.W., McGraw S.M., Niro P.J., Karl J.P., Sonya J.C., Thomas L.C., McClung J.P. (2017). Adherence to the Dietary Guidelines for Americans is associated with psychological resilience in young adults: A cross-sectional study. J. Acad. Nutr. Diet..

[23] Goldstein A.L., Faulkner B., Wekerle C. (2013). The relationship among internal resilience, smoking, alcohol use, and depression symptoms in emerging adults transitioning out of child welfare. Child Abus. Negl..

[24] Tsourtos G., Ward P.R., Miller E.R., Hill K., Barton C., Wilson C.J., Woodman R. (2019). Does resilience moderate the relationship between stress and smoking status?. Subst. Use Misuse..

[25] Hegberg N.J., Tone E.B. (2015). Physical activity and stress resilience: Considering those at-risk for developing mental health problems. Ment. Health Phys. Act..

[26] McCuistion T. (2016). The Relationship between Resilience and Sleep Quality. Ph.D. Thesis.

[27] Hughes J.M., Ulmer C.S., Hastings S.N., Gierisch J.M., Workgroup M.A.V.M., Howard M.O. (2018). Sleep, resilience, and psychological distress in United States military Veterans. Mil. Psychol..

[28] Coronado P.J., Oliva A., Fasero M., Piñel C., Herraiz M.A., Pérez-López F.R. (2015). Resilience and related factors in urban, mid-aged Spanish women. Climacteric.

[29] Kim J.H., Lewis T.T., Topel M.L., Mubasher M., Li C., Vaccarino V., Mujahid M.S., Sims M., Quyyumi A.A., Taylor H.A. (2019). Identification of resilient and at-risk neighborhoods for cardiovascular disease among black residents: The morehouse-emory cardiovascular (MECA) center for health equity study. Prev. Chronic Dis..

[30] Saban K.L., Tell D., Janusek L. (2019). Resilience in African American women at risk for cardiovascular disease: An exploratory study. J. Urban Health.

[31] Chmitorz A., Chmitorz A., Kunzler A., Helmreich I., Tüscher O., Kalisch R., Kubiak T., Wessa M., Lieb K. (2018). Intervention studies to foster resilience—A systematic review and proposal for a resilience framework in future intervention studies. Clin. Psychol. Rev..

[32] Kalisch R., Müller M.B., Tüscher O. (2015). A conceptual framework for the neurobiological study of resilience. Behav. Brain Sci..

[33] Woods-Giscombé C.L., Black A.R. (2010). Mind-body interventions to reduce risk for health disparities related to stress and strength among African American women: The potential of mindfulness-based stress reduction, loving-kindness, and the NTU therapeutic framework. Complement. Health Pract. Rev..

[34] Beal F.M. (2008). Double jeopardy: To be black and female. Meridians.

[35] Panizza C.E., Shvetsov Y.B., Harmon B.E., Wilkens L.R., Le Marchand L., Haiman C., Reedy J., Boushey C.J. (2018). Testing the predictive validity of the healthy eating index-2015 in the multiethnic cohort: Is the score associated with a reduced risk of all-cause and cause-specific mortality?. Nutrients.

[36] Patterson R.E., Kristal A.R., Tinker L.F., Carter R.A., Bolton M.P., Agurs-Collins T. (1999). Measurement characteristics of the Women’s Health Initiative food frequency questionnaire. Ann. Epidemiol..

[37] Hu E.A., Steffen L.M., Coresh J., Appel L.J., Rebholz C.M. (2019). Abstract P215: Adherence to the healthy eating index-2015 may reduce the risk of incident cardiovascular disease, cardiovascular disease mortality, and all-cause mortality. Circulation.

[38] Reedy J., Lerman J.L., Krebs-Smith S.M., Kirkpatrick S.I., Pannucci T.E., Wilson M.M., Subar A.F., Kahle L.L., Tooze J. (2018). Evaluation of the healthy eating index-2015. J. Acad. Nutr. Diet..

[39] Duncan M.S., Freiberg M.S., Greevy R.A., Kundu S., Vasan R.S., Tindle H.A. (2019). Association of smoking cessation with subsequent risk of cardiovascular disease. Jama.

[40] Powell K.E., King A.C., Buchner D.M., Campbell W.W., DiPietro L., Erickson K.I., Charles H.H., John M.J., Kathleen F.J., Kraus W.E. (2019). The scientific foundation for the physical activity guidelines for americans. J. Phys. Act. Health.

[41] LaCroix A.Z., Bellettiere J., Rillamas-Sun E., Di C., Evenson K.R., Lewis C.E., Buchner D.M., Stefanick M.L., Lee I.M., Rosenberg D.E. (2019). Association of light physical activity measured by accelerometry and incidence of coronary heart disease and cardiovascular disease in older women. JAMA Netw. Open.

[42] LaMonte M.J., Lewis C.E., Buchner D.M., Evenson K.R., Rillamas-Sun E., Di C., Lee I.M., Bellettiere J., Stefanick M.L., Eaton C.B. (2017). Both light intensity and moderate-to-vigorous physical activity measured by accelerometry are favorably associated with cardiometabolic risk factors in older women: The objective physical activity and cardiovascular health (OPACH) study. J. Am. Heart Assoc..

[43] Watson N.F., Badr M.S., Belenky G., Bliwise D.L., Buxton O.M., Buysse D., Dinges D.F., Gangwisch J., Grandner M.A., Consensus Conference Panel (2015). Joint consensus statement of the American Academy of sleep medicine and sleep research society on the recommended amount of sleep for a healthy adult: Methodology and discussion. Sleep.

[44] Sands-Lincoln M., Loucks E.B., Lu B., Carskadon M.A., Sharkey K., Stefanick M.L., Ockene J., Shah N., Hairston K.G., Robinson J.G. (2013). Sleep duration, insomnia, and coronary heart disease among postmenopausal women in the Women’s Health Initiative. J. Women Health..

[45] Blow F.C., Barry K.L. (2002). Use and misuse of alcohol among older women. Alcohol Res. Health.

[46] Fernandez-Sola J. (2015). Cardiovascular risks and benefits of moderate and heavy alcohol consumption. Nat. Rev. Cardiol..

[47] Colpani V., Baena C.P., Jaspers L., Van Dijk G.M., Farajzadegan Z., Dhana K., Tielemans M.J., Voortman T., Freak-Poli R., Veloso G.G. (2018). Lifestyle factors, cardiovascular disease and all-cause mortality in middle-aged and elderly women: A systematic review and meta-analysis. Eur. J. Epidemiol..

[48] Smith B.W., Epstein E.M., Ortiz J.A., Christopher P.J., Tooley E.M. (2013). The foundations of resilience: What are the critical resources for bouncing back from stress? in Resilience in children, adolescents, and adults. Resil. Child. Adolesc. Adults..

[49] Woods N.F., Rillamas-Sun E., Cochrane B.B., La Croix A.Z., Seeman T.E., Tindle H.A., Zaslavsky O., Bird C.E., Johnson K.C., Manson J.E. (2016). Aging well: Observations from the women’s health initiative study. J. Gerontol. Ser. A Biomed. Sci. Med Sci..

[50] Ruberman W., Weinblatt E., Goldberg J.D., Chaudhary B.S. (1984). Psychosocial influences on mortality after myocardial infarction. N. Engl. J. Med..

[51] Burnam M.A., Wells K.B., Leake B., Landsverk J. (1988). Development of a brief screening instrument for detecting depressive disorders. Med Care..

[52] Weissman M.M., Sholomskas D., Pottenger M., Prusoff B.A., Locke B.Z. (1977). Assessing depressive symptoms in five psychiatric populations: A validation study. Am. J. Epidemiol..

[53] Silverman A.L., Herzog A.A., Silverman D.I. (2019). Hearts and minds: Stress, anxiety, and depression: Unsung risk factors for cardiovascular disease. Cardiol. Rev..

[54] Olsson L., Jerneck A., Thoren H., Persson J., O’Byrne D. (2015). Why resilience is unappealing to social science: Theoretical and empirical investigations of the scientific use of resilience. Sci. Adv..

[55] Stephens L.D., McNaughton S.A., Crawford D., MacFarlane A., Ball K. (2011). Correlates of dietary resilience among socioeconomically disadvantaged adolescents. Eur. J. Clin. Nutr..

[56] Vesnaver E., Keller H.H., Payette H., Shatenstein B. (2012). Dietary resilience as described by older community-dwelling adults from the NuAge study “If there is a will–there is a way!”. Appetite.

[57] Silverman N.M., Deuster P.A. (2014). Biological mechanisms underlying the role of physical fitness in health and resilience. Interface Focus..

[58] Klaperski S., von Dawans B., Heinrichs M., Fuchs R. (2013). Does the level of physical exercise affect physiological and psychological responses to psychosocial stress in women?. Psychol. Sport Exerc..

[59] Palagini L., Moretto U., Novi M., Masci I., Caruso D., Drake C.L., Riemann D. (2018). Lack of resilience is related to stress-related sleep reactivity, hyperarousal, and emotion dysregulation in insomnia disorder. J. Altern. Complement. Med..

[60] Kemper K.J., Mo X., Khayat R. (2015). Are mindfulness and self-compassion associated with sleep and resilience in health professionals?. J. Altern. Complement. Med..

[61] Ford E.S., Cunningham T.J., Croft J.B. (2015). Trends in self-reported sleep duration among US adults from 1985 to 2012. Sleep.

[62] Slopen N., Lewis T.T., Williams D.R. (2016). Discrimination and sleep: A systematic review. Sleep Med..

[63] St-Onge M.-P., Zuraikat F.M. (2019). Reciprocal roles of sleep and diet. in cardiovascular health: A review of recent evidence and a potential mechanism. Curr. Atheroscler. Rep..

[64] Mesas A.E., Hagen E.W., Peppard P.E. (2018). The bidirectional association between physical activity and sleep in middle-aged and older adults: A prospective study based on polysomnography. Sleep.

[65] Woods-Giscombé C.L. (2010). Superwoman schema: African American women’s views on stress, strength, and health. Qual. Health Res..

[66] Nadal K.L., Mazzula S.L., Rivera D.P., Fujii-Doe W. (2014). Microaggressions and Latina/o Americans: An analysis of nativity, gender, and ethnicity. J. Lat. Psychol..

[67] Watson-Singleton N.N. (2017). Strong black woman schema and psychological distress: The mediating role of perceived emotional support. J. Black Psychol..

[68] Beauboeuf-Lafontant T. (2007). You have to show strength: An. exploration of gender, race, and depression. Gend. Soc..

[69] Woods-Giscombe C.L., Allen A.M., Black A.R., Steed T.C., Li Y., Lackey C. (2019). The Giscombe superwoman schema questionnaire: Psychometric properties and associations with mental health and health behaviors in African American women. Issues Ment. Health Nurs..

[70] Alger S.E., Brager A.J., Capaldi V.F. (2019). Challenging the stigma of workplace napping. Sleep.

[71] Coronado P.J., Fasero M., Oliva A., Piñel C., Herraiz M.A., Pérez-López F.R. (2015). Cross-sectional assessment of resilience in peri-and postmenopausal Spanish women. Maturitas.

[72] Rajpathak S.N., Freiberg M.S., Wang C., Wylie-Rosett J., Wildman R.P., Rohan T.E., Robinson J.G., Liu S., Wassertheil-Smoller S. (2010). Alcohol consumption and the risk of coronary heart disease in postmenopausal women with diabetes: Women’s health initiative observational study. Eur. J. Nutr..

[73] Greve W., Staudinger U.M., Cicchetti D., Cohen A. (2006). Resilience in later adulthood and old age: Resources and potentials for successful aging. Developmental Psychopathology.

