# Supplementary material for: Resilience and CVD-protective Health Behaviors in Older Women: Examining Racial and Ethnic Differences in a Cross-Sectional Analysis of the Women’s Health Initiative
Source: Nutrients. 2020 Jul 16;12(7):2107. doi: 10.3390/nu12072107 (PMC7400950; doi:10.3390/nu12072107)
Supplement: Supplementary file 1 [file nutrients-12-02107-s001.pdf]

Supplemental Materials:

Table 1. Results from full model including interaction between race/ethnicity and resilience.

Outcome	Race/Ethnicity Category	Resilience Category	OR (95% CI)*	P-Value**
Non-smoker (y/n)				
	Black or African American	Low	Ref	0.80
	Black or African American	Medium	0.79 (0.45, 1.39)	
	Black or African American	High	0.93 (0.53, 1.63)	
	Non-Hispanic white	Low	1.57 (0.90, 2.75)	
	Non-Hispanic white	Medium	1.66 (0.98, 2.80)	
	Non-Hispanic white	High	1.68 (0.99, 2.85)	
	Asian or Pacific Islander	Low	3.32 (0.43, 25.59)	
	Asian or Pacific Islander	Medium	3.58 (1.54, 8.34)	
	Asian or Pacific Islander	High	3.11 (1.29, 7.50)	
	Hispanic/Latina	Low	3.17 (0.90, 11.13)	
	Hispanic/Latina	Medium	1.49 (0.79, 2.80)	
	Hispanic/Latina	High	1.58 (0.82, 3.05)	
Moderate alcohol use (had alcoholic drink(s) 1–7 time(s) per week, y/n)	Black or African American	Low	Ref	0.77
	Black or African American	Medium	1.11 (0.80, 1.55)	
	Black or African American	High	1.08 (0.78, 1.50)	
	Non-Hispanic white	Low	2.36 (1.73, 3.23)	
	Non-Hispanic white	Medium	2.86 (2.10, 3.88)	
	Non-Hispanic white	High	3.05 (2.25, 4.14)	
	Asian or Pacific Islander	Low	0.35 (0.16, 0.78)	
	Asian or Pacific Islander	Medium	0.52 (0.36, 0.75)	
	Asian or Pacific Islander	High	0.62 (0.43, 0.91)	
	Hispanic/Latina	Low	1.36 (0.83, 2.25)	
	Hispanic/Latina	Medium	1.51 (1.07, 2.15)	
	Hispanic/Latina	High	1.74 (1.23, 2.45)	
Hours of sleep (7–9 h, y/n)	Black or African American	Low	Ref	0.03
	Black or African American	Medium	0.89 (0.72, 1.12)	
	Black or African American	High	1.21 (0.97, 1.51)	
	Non-Hispanic white	Low	1.85 (1.50, 2.29)	
	Non-Hispanic white	Medium	2.14 (1.74, 2.63)	
	Non-Hispanic white	High	2.55 (2.08, 3.14)	
	Asian or Pacific Islander	Low	0.49 (0.30, 0.79)	
	Asian or Pacific Islander	Medium	0.84 (0.66, 1.08)	
	Asian or Pacific Islander	High	0.82 (0.64, 1.06)	
	Hispanic/Latina	Low	1.56 (1.08, 2.25)	
	Hispanic/Latina	Medium	1.54 (1.20, 1.96)	
	Hispanic/Latina	High	1.70 (1.32, 2.18)	

Physical activity (≥150 min per week, y/n)	Black or African American	Low	Ref	0.22
	Black or African American	Medium	1.05 (0.82, 1.35)	
	Black or African American	High	1.19 (0.93, 1.52)	
	Non-Hispanic white	Low	0.94 (0.74, 1.20)	
	Non-Hispanic white	Medium	1.17 (0.93, 1.48)	
	Non-Hispanic white	High	1.51 (1.20, 1.90)	
	Asian or Pacific Islander	Low	1.12 (0.68, 1.83)	
	Asian or Pacific Islander	Medium	1.36 (1.03, 1.78)	
	Asian or Pacific Islander	High	1.76 (1.33, 2.34)	
	Hispanic/Latina	Low	1.14 (0.77, 1.68)	
	Hispanic/Latina	Medium	1.40 (1.07, 1.84)	
	Hispanic/Latina	High	1.60 (1.22, 2.10)	
Diet quality (top two quintiles, y/n)	Black or African American	Low	Ref	0.94
	Black or African American	Medium	1.15 (0.89, 1.47)	
	Black or African American	High	1.34 (1.05, 1.72)	
	Non-Hispanic white	Low	1.13 (0.89, 1.43)	
	Non-Hispanic white	Medium	1.22 (0.97, 1.54)	
	Non-Hispanic white	High	1.36 (1.08, 1.72)	
	Asian or Pacific Islander	Low	0.80 (0.48, 1.31)	
	Asian or Pacific Islander	Medium	0.89 (0.67, 1.16)	
	Asian or Pacific Islander	High	1.02 (0.77, 1.35)	
	Hispanic/Latina	Low	0.86 (0.57, 1.31)	
	Hispanic/Latina	Medium	0.89 (0.68, 1.17)	
	Hispanic/Latina	High	1.10 (0.83, 1.44)	

*odds ratio with 95% confidence interval **corresponds to test of whether there is effect modification by race/ethnicity.

Table 2. Sensitivity analysis: estimated associations between self-reported psychological resilience and CVD-protective health behaviors, adjusted for stressful life events (SLEs), sociodemographic information (age, race/ethnicity, and education), depression and additional CVD-protective risk factors (BMI, diabetes, hypertension, and dyslipidemia).

**title**	**SLEs**
**Outcome**	**Sociodemographic, depression**<br>**Additional CVD risk factors**<br>**Model 3**
Diet quality (top 2 quintiles, y/n)	**BRS medium:**<br>1.06 (1.00, 1.13)<br>**BRS high:**<br>1.18 (1.11, 1.26)
Non-smoker (y/n)	**BRS medium:**<br>0.88 (0.73, 1.06)<br>**BRS high:**<br>0.87 (0.71, 1.06)
Physical activity (≥150 min per week, y/n)	**BRS medium:**<br>1.14 (1.07, 1.22)

	**BRS high:** 1.42 (1.33, 1.51)
Hours of sleep (7–9 h, y/n)	**BRS medium:** 1.05 (0.98, 1.11)
	**BRS high:** 1.21 (1.14, 1.29)
Alcohol intake (had alcoholic drink(s) 1–7 time(s) per week, y/n)	**BRS medium:** 1.16 (1.09, 1.24)
	**BRS high:** 1.23 (1.15, 1.32)

Table 3. Sensitivity analysis: results from full model including interaction between race/ethnicity and resilience.

Outcome	Race/Ethnicity Category	Resilience Category	OR (95% CI)	P-Value
Non-smoker (y/n)				
	Black or African American	Low	Ref	0.92
	Black or African American	Medium	0.75 (0.42, 1.35)	
	Black or African American	High	0.83 (0.46, 1.49)	
	Non-Hispanic white	Low	1.71 (0.95, 3.07)	
	Non-Hispanic white	Medium	1.57 (0.91, 2.72)	
	Non-Hispanic white	High	1.53 (0.88, 2.66)	
	Asian or Pacific Islander	Low	3.09 (0.40, 23.94)	
	Asian or Pacific Islander	Medium	3.32 (1.39, 7.94)	
	Asian or Pacific Islander	High	2.98 (1.23, 7.22)	
	Hispanic/Latina	Low	2.97 (0.80, 11.00)	
	Hispanic/Latina	Medium	1.42 (0.73, 2.74)	
	Hispanic/Latina	High	1.44 (0.73, 2.84)	
Moderate alcohol use (had alcoholic drink(s) 1–7 time(s) per week, y/n)	Black or African American	Low	Ref	0.77
	Black or African American	Medium	1.08 (0.78, 1.49)	
	Black or African American	High	1.04 (0.76, 1.44)	
	Non-Hispanic white	Low	2.39 (1.76, 3.24)	
	Non-Hispanic white	Medium	2.79 (2.06, 3.76)	
	Non-Hispanic white	High	2.95 (2.18, 3.98)	
	Asian or Pacific Islander	Low	0.34 (0.16, 0.76)	
	Asian or Pacific Islander	Medium	0.49 (0.34, 0.71)	
	Asian or Pacific Islander	High	0.60 (0.41, 0.86)	
	Hispanic/Latina	Low	1.36 (0.83, 2.22)	
	Hispanic/Latina	Medium	1.48 (1.05, 2.08)	
	Hispanic/Latina	High	1.67 (1.19, 2.35)	
Hours of sleep (7–9 h, y/n)	Black or African American	Low	Ref	0.03
	Black or African American	Medium	0.84 (0.67, 1.05)	
	Black or African American	High	1.10 (0.88, 1.37)	
	Non-Hispanic white	Low	1.92 (1.55, 2.37)	
	Non-Hispanic white	Medium	2.03 (1.65, 2.49)	
	Non-Hispanic white	High	2.35 (1.91, 2.88)	
	Asian or Pacific Islander	Low	0.46 (0.28, 0.75)	

	Asian or Pacific Islander	Medium	0.79 (0.62, 1.02)	
	Asian or Pacific Islander	High	0.76 (0.59, 0.98)	
	Hispanic/Latina	Low	1.58 (1.09, 2.28)	
	Hispanic/Latina	Medium	1.47 (1.15, 1.87)	
	Hispanic/Latina	High	1.56 (1.22, 2.00)	
Physical activity (≥150 min per week, y/n)	Black or African American	Low	Ref	0.26
	Black or African American	Medium	1.00 (0.78, 1.28)	
	Black or African American	High	1.10 (0.86, 1.40)	
	Non-Hispanic white	Low	0.98 (0.77, 1.23)	
	Non-Hispanic white	Medium	1.13 (0.90, 1.41)	
	Non-Hispanic white	High	1.41 (1.12, 1.77)	
	Asian or Pacific Islander	Low	1.04 (0.63, 1.69)	
	Asian or Pacific Islander	Medium	1.29 (0.99, 1.69)	
	Asian or Pacific Islander	High	1.66 (1.25, 2.19)	
	Hispanic/Latina	Low	1.16 (0.78, 1.72)	
	Hispanic/Latina	Medium	1.34 (1.03, 1.76)	
	Hispanic/Latina	High	1.50 (1.15, 1.96)	
Diet quality (top two quintiles, y/n)	Black or African American	Low	Ref	0.93
	Black or African American	Medium	1.14 (0.89, 1.46)	
	Black or African American	High	1.32 (1.03, 1.69)	
	Non-Hispanic white	Low	1.14 (0.90, 1.45)	
	Non-Hispanic white	Medium	1.21 (0.96, 1.52)	
	Non-Hispanic white	High	1.34 (1.06, 1.69)	
	Asian or Pacific Islander	Low	0.79 (0.48, 1.29)	
	Asian or Pacific Islander	Medium	0.88 (0.67, 1.16)	
	Asian or Pacific Islander	High	1.00 (0.76, 1.32)	
	Hispanic/Latina	Low	0.87 (0.58, 1.32)	
	Hispanic/Latina	Medium	0.89 (0.67, 1.17)	
	Hispanic/Latina	High	1.08 (0.82, 1.42)	

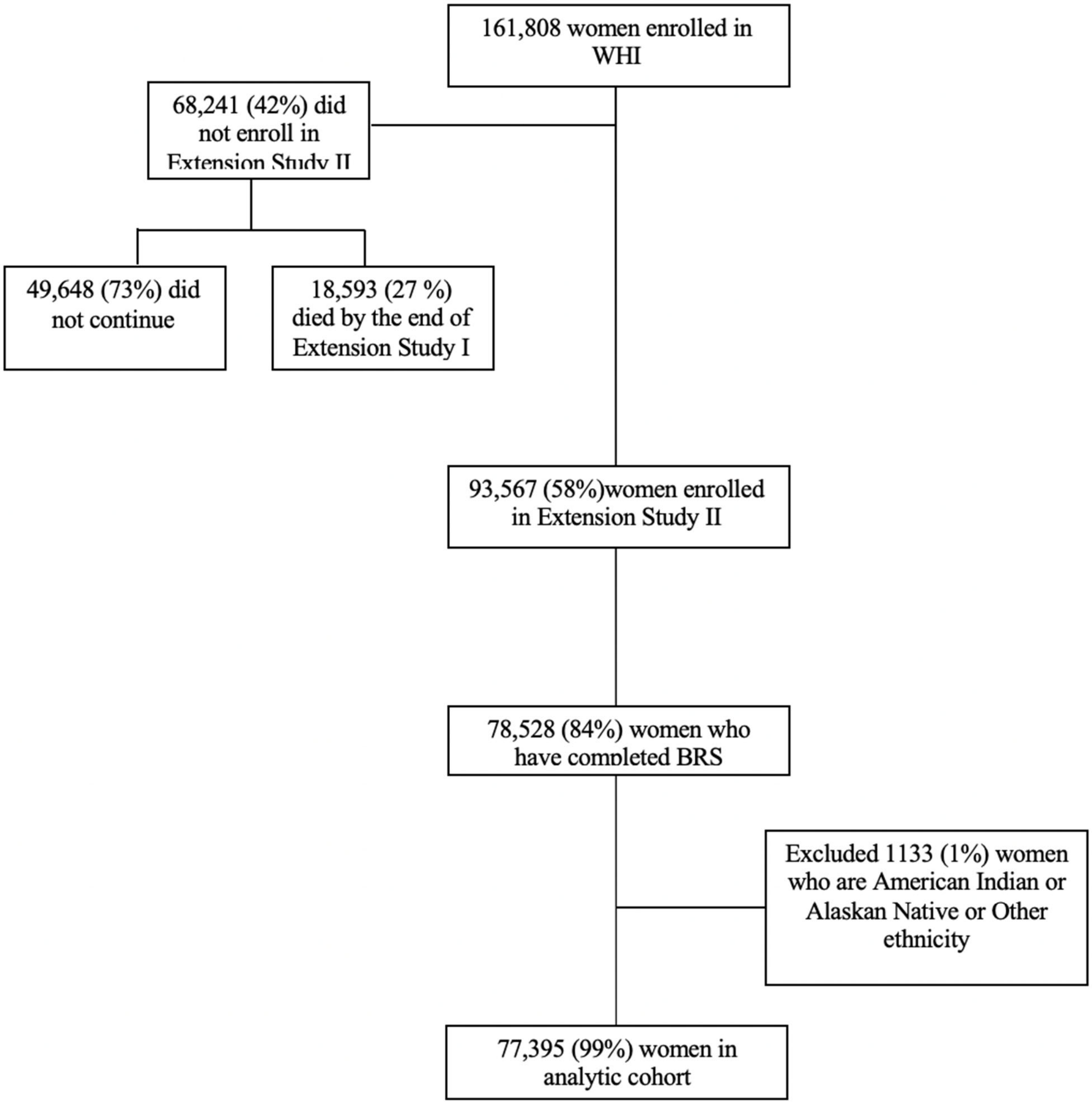

Figure S1: Study flowchart illustrating participant dropout.

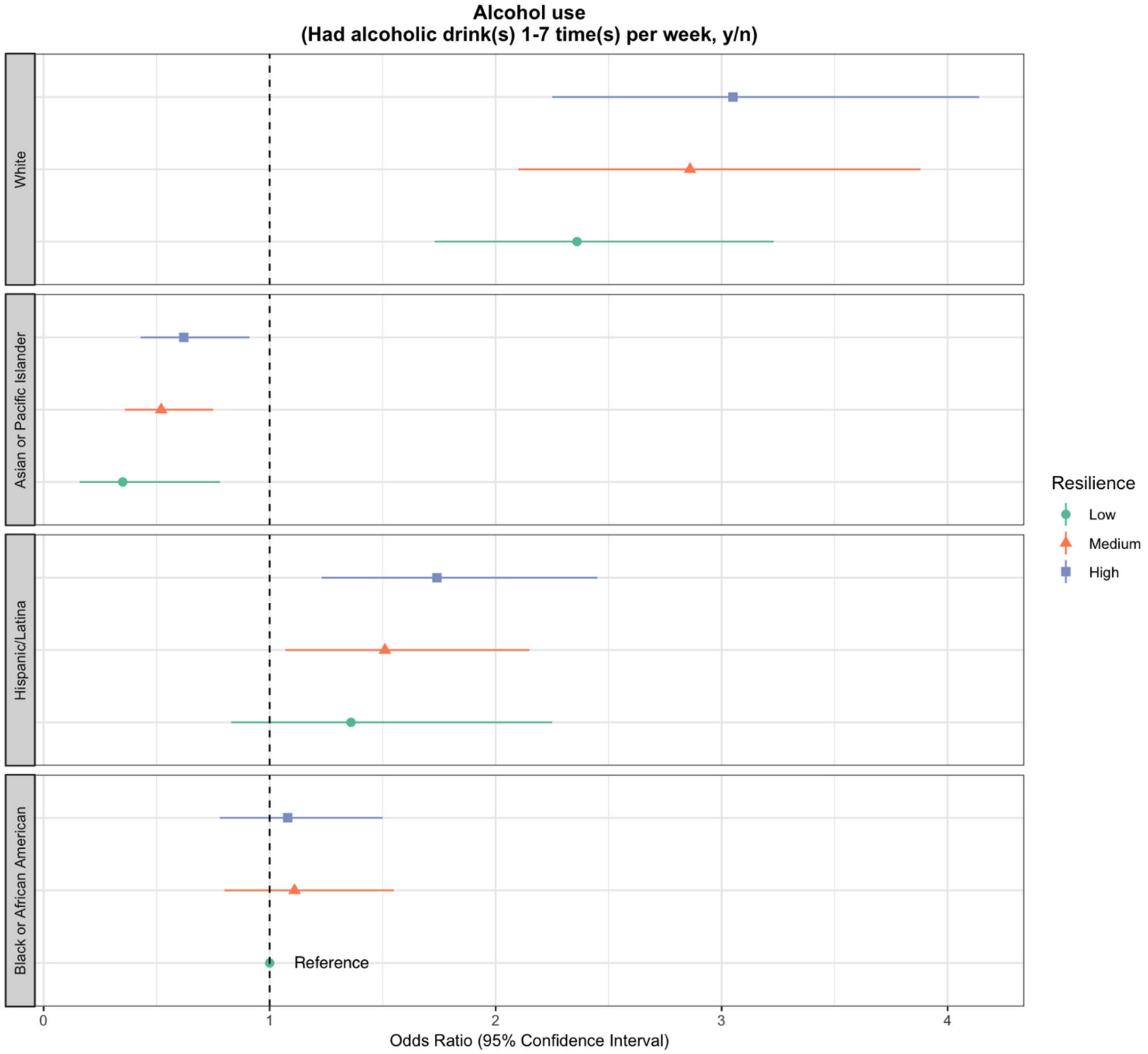

Figure 2. Estimated associations between self-reported psychological resilience and moderate alcohol use, multivariable linear regression adjusted for stressful life events (SLEs), sociodemographic factors (age, race/ethnicity, and education), additional CVD-protective risk factors (BMI, diabetes, hypertension, and high cholesterol), and interaction between race/ethnicity and resilience.

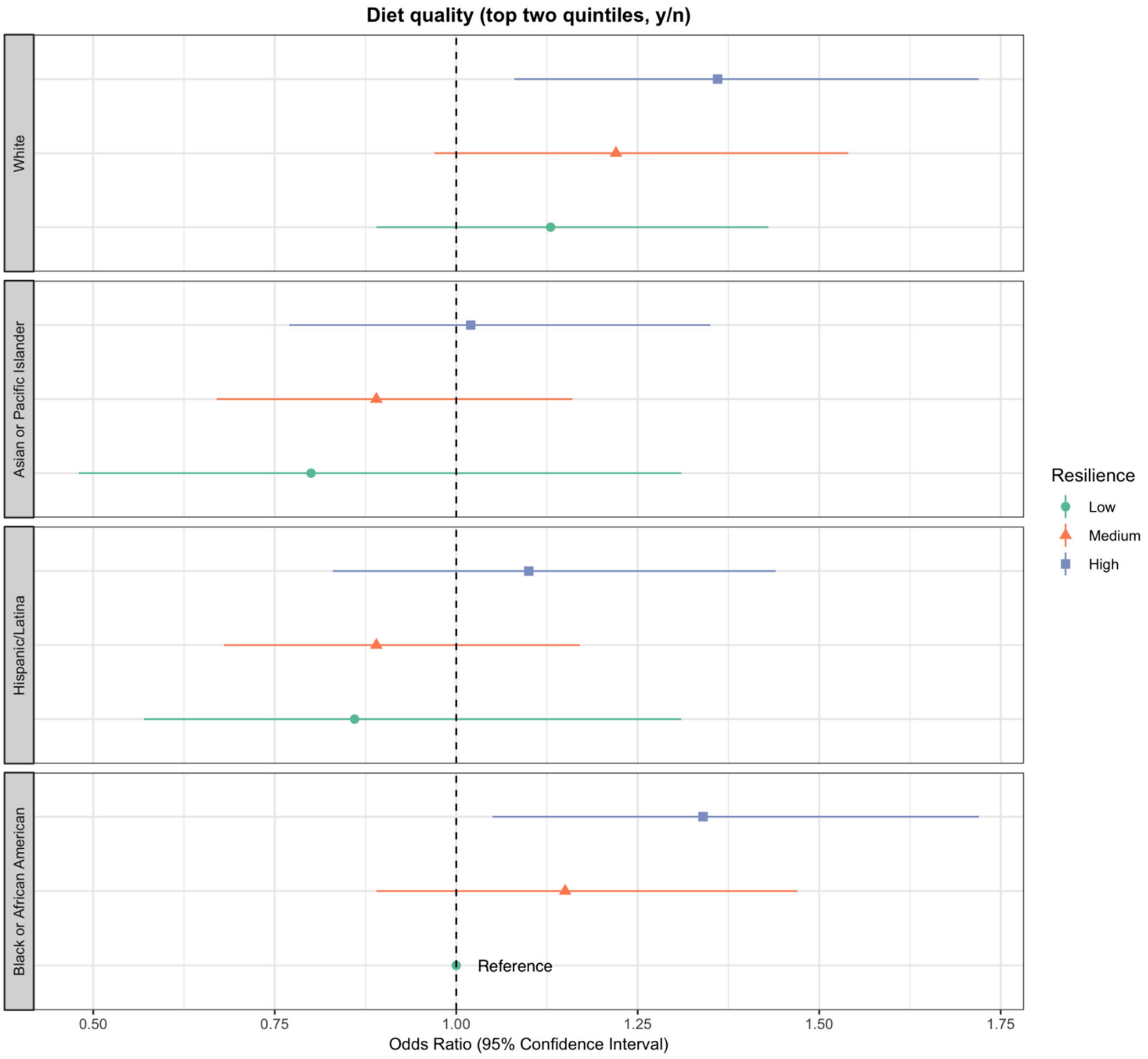

Figure 3. Estimated associations between self-reported psychological resilience and better diet quality, multivariable linear regression adjusted for stressful life events (SLEs), sociodemographic factors (age, race/ethnicity, and education), additional CVD-protective risk factors (BMI, diabetes, hypertension, and high cholesterol), and interaction between race/ethnicity and resilience.

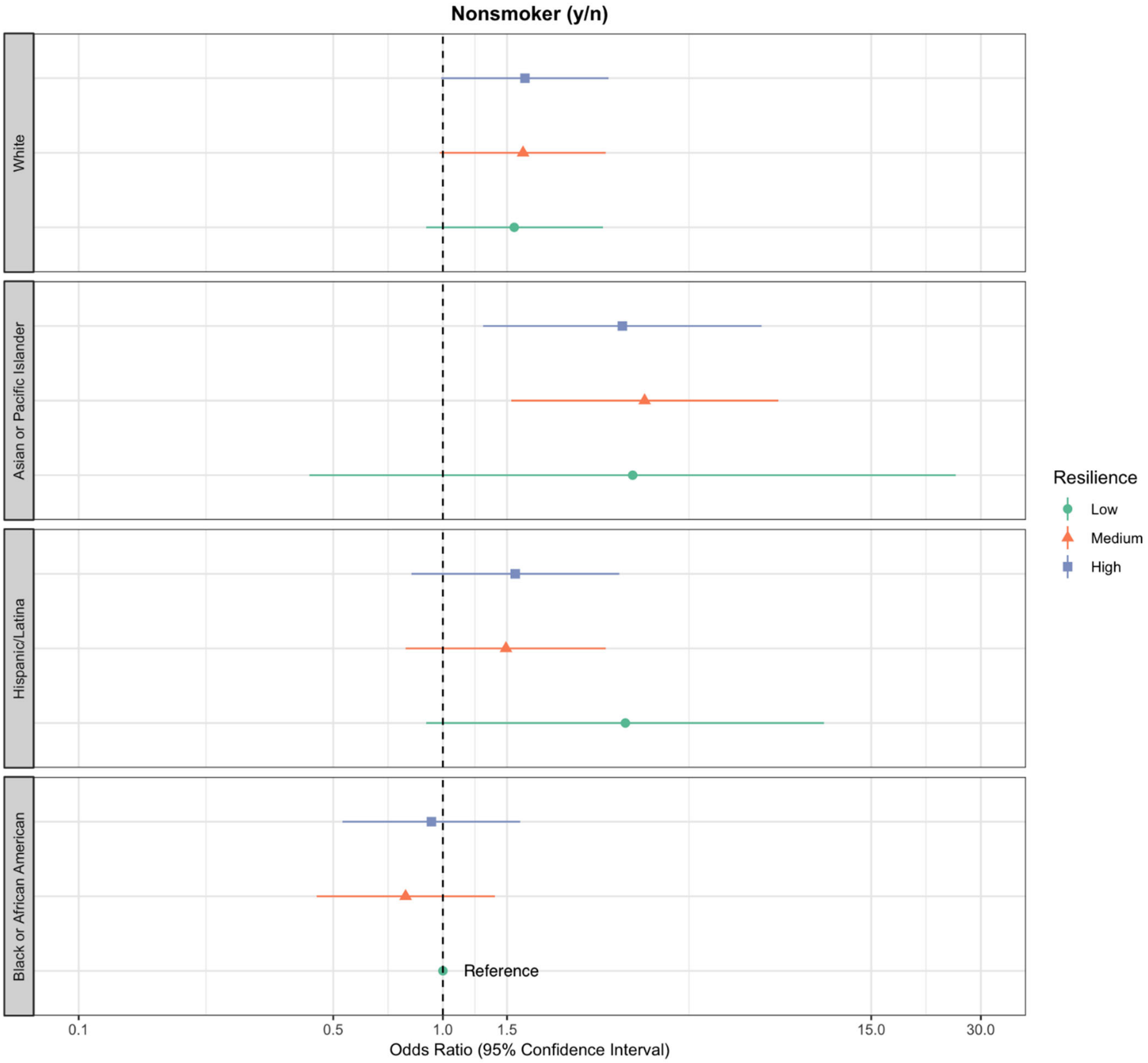

Figure 4. Estimated associations between self-reported psychological resilience and being current non-smoker, multivariable linear regression adjusted for stressful life events (SLEs), sociodemographic factors (age, race/ethnicity, and education), additional CVD-protective risk factors (BMI, diabetes, hypertension, and high cholesterol), and interaction between race/ethnicity and resilience.

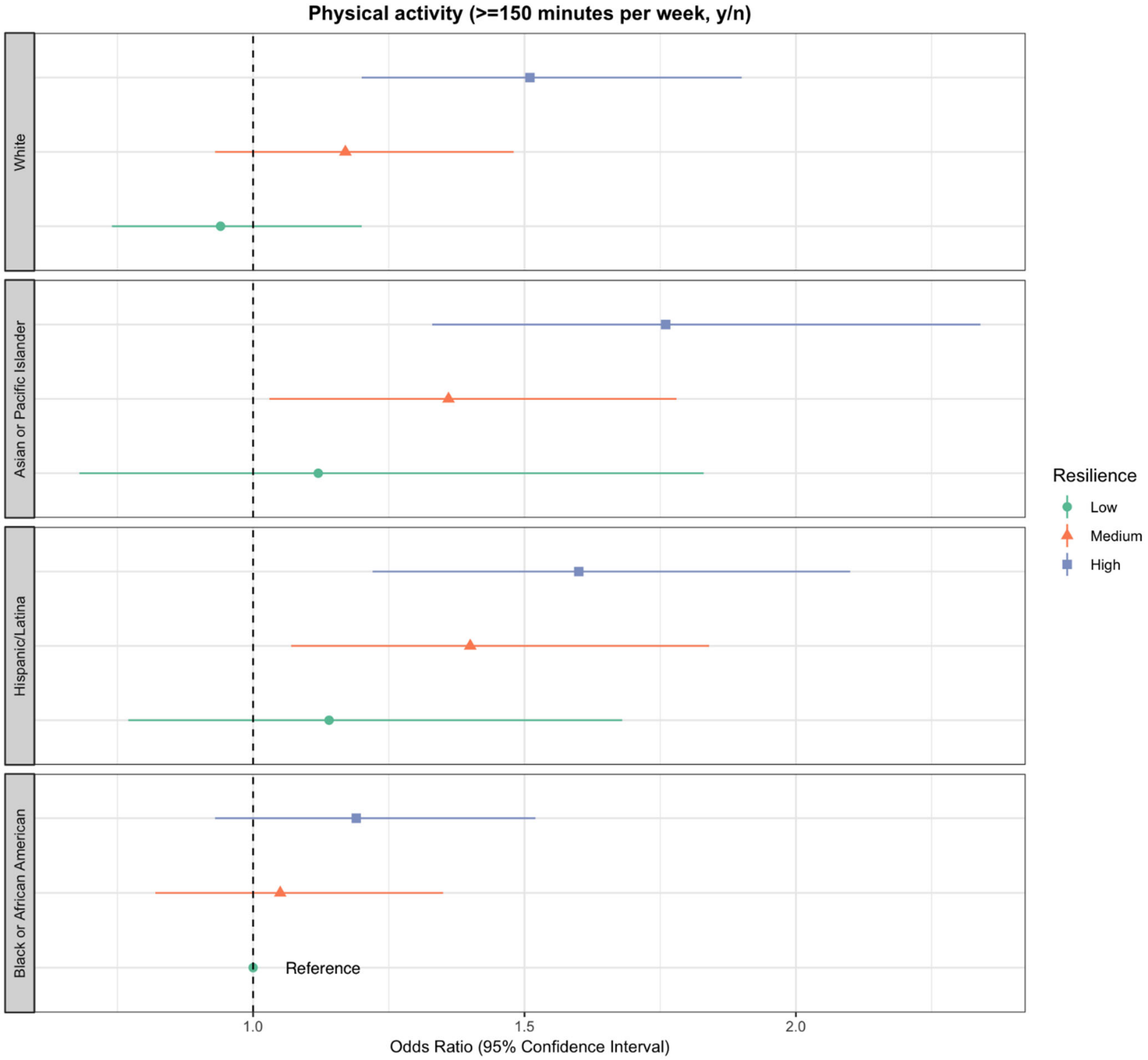

Figure 5. Estimated associations between self-reported psychological resilience and recommended physical activity, multivariable linear regression adjusted for stressful life events (SLEs), sociodemographic factors (age, race/ethnicity, and education), additional CVD-protective risk factors (BMI, diabetes, hypertension, and high cholesterol), and interaction between race/ethnicity and resilience.
